# Predicting in vivo absorption of chloramphenicol in frogs using in vitro percutaneous absorption data

**DOI:** 10.1186/s12917-021-02765-5

**Published:** 2021-01-28

**Authors:** Victoria K. Llewelyn, Lee Berger, Beverley D. Glass

**Affiliations:** 1 Pharmacy, College of Medicine and Dentistry, James Cook University, Townsville, Australia; 2 College of Nursing and Health Sciences, Flinders University, Adelaide, Australia; 3 One Health Research Group, Melbourne Veterinary School, University of Melbourne, Werribee, Australia

**Keywords:** Frog, Disease, Treatment, Chytridiomycosis, Transdermal, Skin absorption

## Abstract

**Background:**

Infectious disease, particularly the fungal disease chytridiomycosis (caused by *Batrachochytrium dendrobatidis*), is a primary cause of amphibian declines and extinctions worldwide. The transdermal route, although offering a simple option for drug administration in frogs, is complicated by the lack of knowledge regarding percutaneous absorption kinetics. This study builds on our previous studies in frogs, to formulate and predict the percutaneous absorption of a drug for the treatment of infectious disease in frogs. Chloramphenicol, a drug with reported efficacy in the treatment of infectious disease including *Batrachochytrium dendrobatidis*, was formulated with 20% v/v propylene glycol and applied to the ventral pelvis of *Rhinella marina* for up to 6 h. Serum samples were taken during and up to 18 h following exposure, quantified for chloramphenicol content, and pharmacokinetic parameters were estimated using non-compartmental analysis.

**Results:**

Serum levels of chloramphenicol reached the minimum inhibitory concentration (MIC; 12.5 μg.mL^− 1^) for *Batrachochytrium dendrobatidis* within 90–120 min of exposure commencing, and remained above the MIC for the remaining exposure time. C_max_ (17.09 ± 2.81 μg.mL^− 1^) was reached at 2 h, while elimination was long (t_1/2_ = 18.68 h).

**Conclusions:**

The model, based on in vitro data and adjusted for formulation components and in vivo data, was effective in predicting chloramphenicol flux to ensure the MIC for *Batrachochytrium dendrobatidis* was reached, with serum levels being well above the MICs for other common bacterial pathogens in frogs. Chloramphenicol’s extended elimination means that a 6-h bath may be adequate to maintain serum levels for up to 24 h. We suggest trialling a reduction of the currently-recommended continuous (23 h/day for 21–35 days) chloramphenicol bathing for chytrid infection with this formulation.

**Supplementary Information:**

The online version contains supplementary material available at 10.1186/s12917-021-02765-5.

## Background

Infectious disease in frogs is a primary cause of population declines worldwide [1]. In particular, the chytrid fungus *Batrachochytrium dendrobatidis* has caused mass declines and extinctions in many frog species [2, 3]. Despite numerous reports of treatment options for *Batrachochytrium dendrobatidis* infection (for review, see [4], and also [5–13]), no consistently effective treatment has been identified across frog species. Further, the wide distribution and highly transmissible nature of *Batrachochytrium dendrobatidis* makes broad-scale treatment of infection in the wild impracticable, and mitigation remains a challenge. Thus, collection and maintenance of disease-free captive breeding and insurance colonies remain key for conservation [14, 15].

Chloramphenicol is a broad-spectrum antibiotic with bacteriostatic activity against a wide variety of organisms including streptococci, staphylococci, gram-negative organisms including *Escherichia coli, Salmonella* spp*.*, anaerobic bacteria, *Mycoplasma* spp*.* and rickettsiae [16], and also has documented activity against chytrid fungi [17]. In captive frogs, a wide variety of bacteria are commonly cultured from sick animals, including *Aeromonas hydrophila*, *Flavobacterium*, enterobacteria including *Citrobacter*, *Proteus* and *Salmonella*, *Mycobacterium, Pseudomonas* spp., *Streptococcus* and *Staphylococcus* [18–21]. Further, frogs with chytridiomycosis often exhibit secondary bacterial infections [22, 23], and so chloramphenicol presents as an ideal drug candidate for treatment of infectious disease in these animals.

Chloramphenicol has been used successfully for treatment of bacterial infection in several different frog species, with a wide variety of doses, administration routes, and treatment durations reported [24–29]. Studies into the use of chloramphenicol for chytridiomycosis specifically have reported mixed results, with different outcomes reported between species and infection severity and inconsistencies in treatment duration [11, 13, 24, 29]. While 20 mg. L^− 1^ chloramphenicol solution bathed continuously for 21–35 days was reported to successfully treat experimentally-infected *Litoria ewingii* and *L. raniformis* Poulter et al. [29], shorter treatment (15 days) in *Pleurodema somuncurense* [13] and up to 28 days in *Rana sphenocephala* [11] reduced infection intensity but did not cure the disease. Conversely, Young et al. [28] reported successful treatment of chytridiomycosis in three terminally ill *L. caerulea* following therapy with heat, electrolyte replacement, and shallow immersion in 20 mg. L^− 1^ chloramphenicol for 23 h daily for a total of 14 days, and complete disease resolution in sub-clinically-infected frogs within 9 days of the same therapy.

The transdermal route for drug administration in frogs provides a rapid and effective alternative to standard dosing routes. This is because frog skin is the primary regulator of fluid and electrolyte levels in the body, providing a highly permeable interface with its immediate environment [30]. However, despite the utility of this route, there is little information available to guide dosing regimen or formulation design for transdermal delivery in frogs. Indeed, doses in frogs are often “scaled down” from those used in mammals, despite the significant anatomical and physiological differences between mammals and amphibians [31]. Some practitioners recommend further adjustments based on basal metabolic rate differences, or allometric scaling when pharmacokinetic differences between species are known [32, 33]. However, the knowledge of pharmacokinetic parameters in frogs is limited, and wide discrepancies between calculated and observed absorption rates have been reported [34]. These issues highlight the need for information to predict percutaneous absorption in frogs.

Recently, we developed in vitro models of absorption in the cane toad (*Rhinella marina*) based on three model chemicals, and determined how well the models’ predictions of absorption matched the in vivo absorption of the same chemicals [35]. We have also reported the impact of penetration enhancers on absorption through frog skin [36]. The broad aim of this study is to determine the utility of these previous results in predicting the in vivo percutaneous absorption of a drug for treatment of infectious disease in frogs. To this end, we: (1) used the results of our previous studies to formulate chloramphenicol as a topical dosage form for use in frogs, and (2) tested the percutaneous absorption of this formulation in healthy frogs in vivo.

## Results

### Bathing solution and urine output

**Fig. 1:**
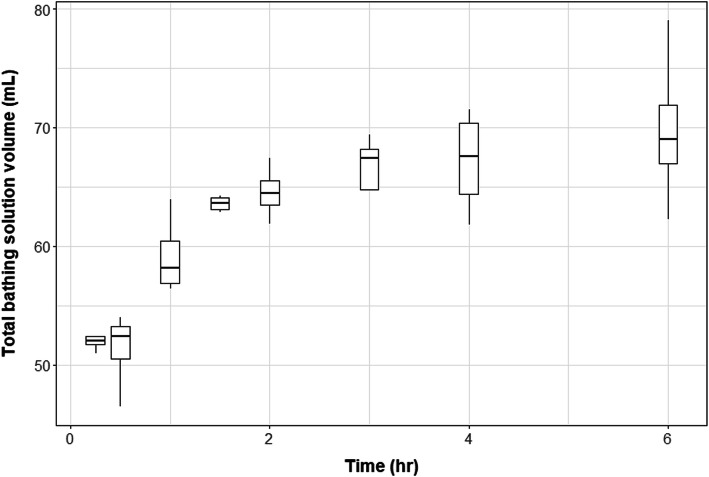
Increases in bathing solution volume due to urine output with increasing exposure time

All animals except one produced urine during the exposure phase. Urine production increased over the exposure period (Fig. 1). No animals produced urine during the elimination phase of the trial. Percentage of chloramphenicol remaining in the dosing solution / urine decreased over the exposure period, to an average drug content of 75.02% at t = 6 h (range 50.45–90.99%). No correlation was observed between urine production and amount of chloramphenicol in the bathing solution.

### Pharmacokinetic study

No noticeable adverse effects were observed in any animals. All non-control animals had quantifiable chloramphenicol levels at all sampling times; chloramphenicol was not detected in control animals.

**Fig. 2:**
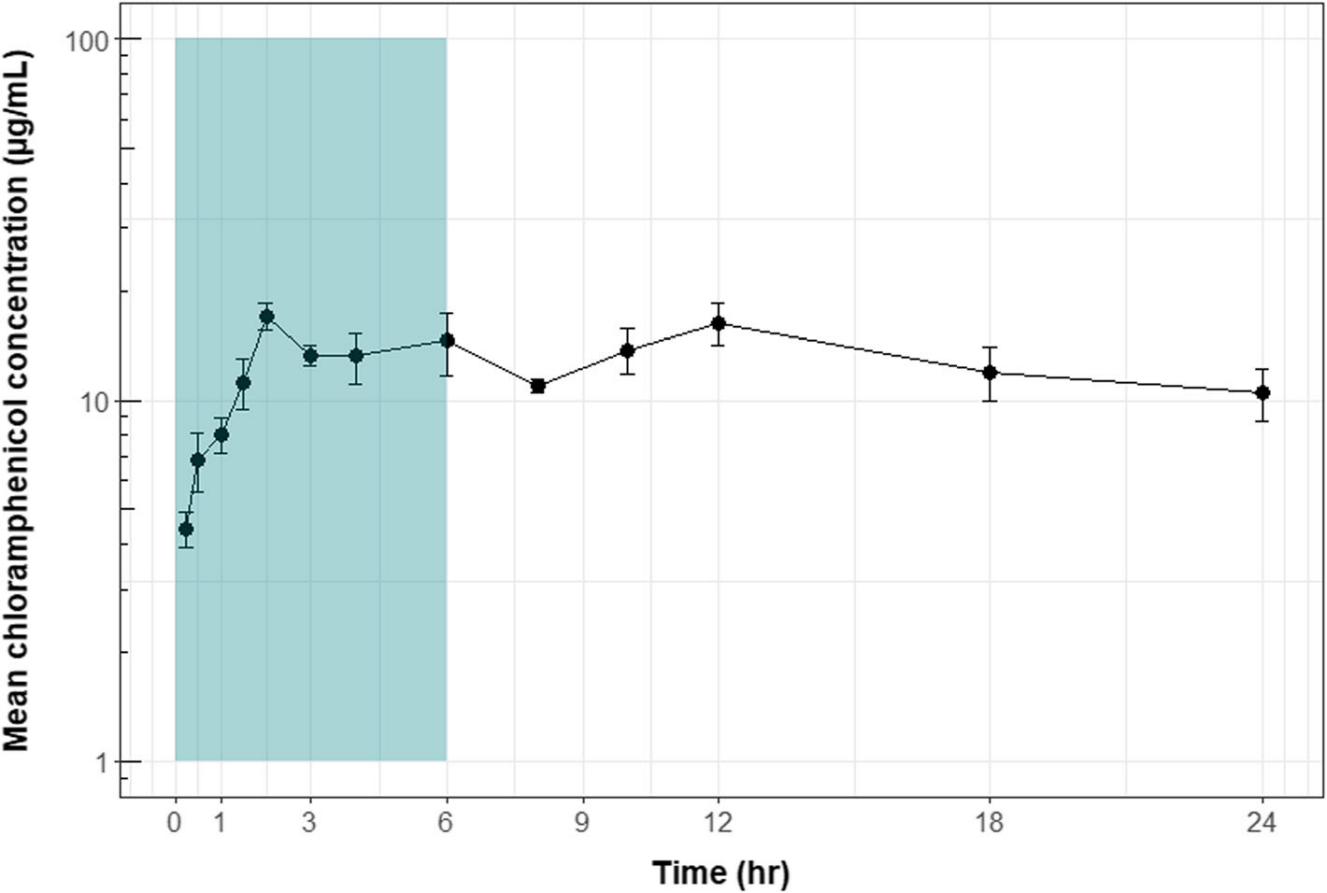
Drug concentration curves (± standard deviation; SD) for chloramphenicol following application to the skin in cane toads. Note: drug exposure period (teal): t = 0 to t = 6 h, elimination period from t = 6 to t = 24 h

In order to investigate the impact of using different-sized animals on absorption profile, concentration-time curves were prepared from raw serum concentration data and also with concentration adjusted for animal weight or body surface area. No significant differences in profiles were noted (data not shown). Thus, the mean serum concentration versus time profile for chloramphenicol following topical administration in cane toads is shown in Fig. 2, and mean concentration values for each time point (*N* = 4 samples per time point) are presented in Table 1. The concentration-time curve shows a biphasic profile, with a secondary peak at t = 12 h, suggesting ongoing distribution of the drug for an extended time following cessation of dosing. Mean levels above the minimum inhibitory concentration (MIC) for *Batrachochytrium dendrobatidis* (12.5 μg.mL^− 1^ [37];) were reached between 1.5 and 2 h after bathing commenced, and remained above this value for the duration of exposure. Pharmacokinetic parameters are presented in Table 2. time to maximum plasma concentration (T_max_) was 2 h, and maximum observed plasma concentration (C_max_) was 17.094 ± 2.813 μg.mL^− 1^.

**Table 1:** Plasma concentrations of chloramphenicol after topical administration to the ventral pelvis in cane toads

	Plasma concentration (μg.mL^**−1**^)	
Sample Time (h)	Mean ± SD	Median (range)
0.25	4.40 ± 0.99	4.34 (3.27–5.64)
0.50	6.84 ± 2.48	7.21 (3.51–9.43)
1.00	8.01 ± 1.79	7.67 (6.31–10.39)
1.50	11.26 ± 3.56	11.84 (7.11–14.26)
2.00	17.09 ± 2.81	17.59 (13.39–19.80)
3.00	13.37 ± 1.74	13.03 (11.86–15.58)
4.00	13.24 ± 4.20	12.08 (9.56–19.25)
6.00	14.61 ± 5.73	12.70 (10.31–22.75)
8.00	11.00 ± 0.94	11.32 (9.63–11.74)
10.00	13.80 ± 4.01	15.72 (7.79–15.97)
12.00	16.36 ± 4.23	17.11 (10.60–20.64)
18.00	11.99 ± 4.00	11.66 (7.72–16.95)
24.00	10.48 ± 3.44	9.75 (7.13–15.30)

Values are reported as mean (± standard deviation; SD) and median plus range. *N* = 4 for all sample times.

**Table 2:** Pharmacokinetic parameters of chloramphenicol following ventral pelvic exposure in cane toads for 6 h

Parameter	Value^**a**^
T_max_ (h)	2
C_max_ (μg.mL^− 1^)	17.094
AUC_0–last_ (μg.h/mL^− 1^)	306.019
AUC_0–∞_ (μg.h/mL^−1^)	588.461
k_el_	0.037
Half-life (h)	18.676

^a^: all values are mean values except T_max_ which is a median value

## Discussion

This study represents the first attempt to use our model of in vitro percutaneous absorption in the cane toad, coupled with previous in vivo percutaneous absorption studies of model chemicals [35], to predict the in vivo absorption of a drug for treatment of infectious disease through frog skin. Serum concentrations of chloramphenicol achieved reached the MIC (12.5 μg.mL^− 1^) required for treatment of *Batrachochytrium dendrobatidis* infection, as predicted by the model. These levels were well in excess of those required for treatment of many other common bacterial pathogens in frogs, including *A. hydrophila, Proteus penneri, Salmonella enterica*, *Streptococcus* spp., and *Staphylococcus aureus* (Table 3). Serum concentrations of chloramphenicol required to treat these bacterial organisms were reached within 15 min of bathing commencing.

**Table 3:** Common bacterial pathogens in frogs and reported minimum inhibitory concentration for chloramphenicol for these pathogens

Organism	MIC (μg.mL^**−1**^)	Reference
*Aeromonas hydrophila*	≤ 0.5–2	[38]
*Citrobacter freundii*	8–> 32	[39]
*Flavobacterium*	Resistant	[40]
*Proteus mirabilis*	7–36	[41]
*Proteus penneri*	1–7	[41]
*Pseudomonas aeruginosa*	32–resistant	[42]
*Salmonella enterica*	0.5–8	[42]
*Staphylococcus aureus*	2–8	[42]
*Streptococcus pneumoniae*	1–4	[43]
*Streptococcus pyogenes*	2–4	[43]

Exposure time (1.5–2 h) required to achieve the target MIC for *Batrachochytrium dendrobatidis* was slightly longer than the model’s prediction of ~ 40 min, however as the model was based on in vitro absorption data, it is unable to account for lag time to steady state nor any other pharmacokinetic parameters that occur in vivo. It is likely that these in vivo processes lengthened the time taken to reach the target MIC.

Pharmacokinetic studies are rare in frogs, particularly those following topical application. This study provides the first preliminary pharmacokinetic data for chloramphenicol in these animals. Despite being unreported in frogs, the pharmacokinetics of chloramphenicol have been documented in many other species, including birds and mammals. Chloramphenicol is typically widely distributed into the tissues and central nervous system, with volume of distribution reported in the range 1–3 L.kg^− 1^ for most animals [16]. Its primary route of elimination is via hepatic glucuronidation, with the inactive metabolite and remaining parent drug being renally excreted. In comparison to the findings in this study, elimination in mammals is typically rapid, with half-life in the range of 1–3 h [44]. The prolonged elimination of chloramphenicol found in this study is likely due to differences in elimination capacity in frogs compared to mammals. In particular, although it appears that frogs have the full complement of hepatic microsomes, the relative activity of these is much lower than in mammals [45]. Further, differences in the structure and function of the urinary system in frogs is also likely to influence the elimination of chloramphenicol. As frogs lack the ability to concentrate their urine [46], lipophilic chemicals are unlikely to be reabsorbed in the renal tubules as occurs in mammals. This, coupled with the decreased hepatic enzyme activity, may suggest that more chloramphenicol will be excreted unchanged in frogs compared to that reported in mammals (6 and 25% in dogs and cats, respectively [44]). However, renal excretion alone could not be measured in the current study, as animals urinated into the bathing solution during the exposure period, and no animals in the elimination period produced urine. As no distinct relationship was noted between urine production and amount of chloramphenicol in the bathing solution, it is likely that in cane toads, both metabolism and urinary excretion contribute to elimination of chloramphenicol — i.e., some chloramphenicol is metabolised, and some excreted unchanged into the bathing solution. Single-dose kinetic studies in frogs will provide clarification of elimination kinetics of chloramphenicol in frogs.

Of particular interest in this study is the slow distribution and elimination of chloramphenicol from the body after cessation of drug administration. In particular, the secondary peak seen at t = 12 h in the current study is unusual. It may be due to chloramphenicol accumulation in the skin forming a depot, or may alternatively reflect a wide distribution to the tissues, with redistribution into the serum occurring after cessation of drug exposure. The finding of extended elimination also has clinical relevance: given the long half-life of chloramphenicol following topical administration, once-daily dosing may be sufficient to maintain therapeutic levels in vivo.

Chloramphenicol has been associated with potentially-fatal blood dyscrasias in humans and other animals, including frogs [47], and its use systemically in humans is limited. Owing to these effects in humans, its use in food-production animals is also prohibited in many regions, including the European Union, Canada, the United States of America, Japan, and Australia [48]. Chloramphenicol is, however, experiencing a resurgence in use in companion animal medicine, owing to its broad spectrum of activity and increasing microbial resistance to other, safer agents [16]. In regards to the occurrence of blood dyscrasias in frogs, it is unlikely that the dose regimen used in the current study would be of concern, as both the dose and duration of chloramphenicol exposure in the current study (6.25 mg/100 g body weight for 6 h) is far lower than that used in the study reporting chloramphenicol-associated leukaemia in toads (4 mg/40 g body weight for three months [47];). However, with increasing use of chloramphenicol and other broad-spectrum antibiotics in the management of infectious disease in frogs, the impact of these on the frog, human and animal microbiome must also be considered. Frog skin has symbiotic bacteria and fungi, many of which have been shown to produce protective secretions. Recently, much research in amphibian microbial ecology has focussed on the effects of these symbiotic bacteria and fungi on *Batrachochytrium dendrobatidis* infection, finding many produce antifungal (including anti-*Batrachochytrium dendrobatidis*) metabolites in culture, and also reporting correlations between the relative abundance of some skin bacteria and *Batrachochytrium dendrobatidis* prevalence [49]. One study has reported on the impact of a 200 μg/ml chloramphenicol solution on a series of bacterial isolates from *R. sphenocephala* skin, finding it significantly inhibited growth in all isolates [11]. Although the isolates were not identified, we suggest that chloramphenicol only be used to treat disease in frogs when the benefits outweigh the risks associated with such perturbation of the skin microbiome, and remind clinicians to be mindful of the alterations to the natural frog microbiome that will occur as a result of such treatment.

A limitation of the current study is that it was performed solely in male animals. As the study is a preliminary one, using a sole gender is preferable to minimise any intergender differences in pharmacokinetics. However, from a pharmacokinetic perspective, using only one gender may skew the pharmacokinetic profile that is presented, as in some animal species there are gender differences in metabolic pathways [50]. Little is known about metabolic pathways in frogs, however, there is evidence of seasonal differences in CYP450 activity between male and female frogs [45].

Finally, note must be made that although the current study also aimed to provide dosing information for treatment of infection in frogs, the study was carried out in healthy animals. Pharmacokinetic processes change when an animal’s normal homeostasis is altered due to disease. In frogs, their ability to excrete drugs in the urine will be substantially reduced if they are dehydrated, as urine production is halted in frogs as a mechanism to preserve water when they are dehydrated [51]. Further, chytridiomycosis infection can alter the skin by causing hyperkeratosis and ulceration [52], which is likely to affect percutaneous owing to changes to the skin’s barrier function. These changes must be considered when extrapolating pharmacokinetic findings in healthy animals to sick animals, and so the results of the current study provide a preliminary basis from which to design effective dosing schedules for treatment of disease in frogs.

## Conclusions

The serum concentrations of chloramphenicol achieved during the exposure time were consistent with the predictions of our model, despite these predictions being based on in vitro absorption data alone, which disregards the parallel processes of distribution and elimination that occur in vivo. A dose of 250 μg.mL^− 1^ chloramphenicol administered to the ventral pelvic patch in cane toads achieved the required MIC for treatment of chytridiomycosis within two hours of commencing bathing, and exceeded the MIC required for several other common bacterial pathogens of frogs within the first 15 min of bathing. Although the current treatment protocol for chytridiomycosis using chloramphenicol requires continuous bathing for 3–5 weeks, our results suggest that 6-hourly baths daily may be sufficient to maintain adequate chloramphenicol levels in vivo. These shorter daily bath times should be investigated, as intermittent bathing would optimise this treatment regimen, making it accessible to frog species that cannot tolerate continuous bathing. Further, such refinement would also improve compliance and utility of this treatment in practice. The models described previously [35] may also be used to select and formulate other drugs for treatment of infectious disease in amphibians, thereby maintaining the health of captive insurance populations and hastening the successful reintroduction of these animals to their original environments.

## Methods

### Chemicals and solutions

Amphibian Ringers solution (ARS) contained: 113 mM sodium chloride, 2 mM potassium chloride, 1.35 mM calcium chloride, 2.4 mM sodium bicarbonate [53]. Chloramphenicol dosing solution was prepared from high-performance liquid chromatography (HPLC)-grade chloramphenicol (≥98%; Sigma) in 20% v/v PG (pharmaceutical grade, Chem-Supply) in ARS solution, to provide a final dose of ~ 250 μg.mL^− 1^. Ethyl 3-aminobenzoate methanesulfonate (MS-222; Aldrich Chemistry) solutions were prepared at two strengths in purified water: 0.2% w/v, buffered to pH 7.3 with sodium bicarbonate [54], and 12.5% w/v, unbuffered [55]. Water used throughout was ultrapure (Milli-Q Integral, Millipore). All solutions were freshly prepared.

Serum extractions used sodium hydroxide 10 mmol solution prepared from reagent-grade sodium hydroxide pellets (Chem-Supply), and ethyl acetate (reagent grade, Ajax Finechem PTY LTD). The internal standard (IS) was carbamazepine (USP testing standard, Sigma), prepared at a concentration of 500 μg.mL^− 1^ in HPLC-grade methanol (Fisher Chemicals, Trinidad and Thermo Fisher Scientific). The HPLC mobile phase used methanol (HPLC grade; Fisher Chemicals, Trinidad and Thermo Fisher Scientific) and ultrapure water (Milli-Q Integral, Millipore), acidified with 0.2% v/v acetic acid (ACS reagent grade; Lab-Scan).

### Animal husbandry

Fifty-five adult male cane toads (*Rhinella marina*), weighing 75.8–140.1 g (mean 104.1 g) were wild-caught in the Townsville region (Australia). Cane toads were used as this species is the same as was used in previous in vitro and in vivo studies to produce the model used to predict absorption in this study [35]. Animals were transported to the laboratory and housed in groups of 3 or 4 in plastic tubs (60 × 36 × 40 cm) in a dedicated room maintained at 21 ± 2 °C. The base of each tub was lined with absorbent paper, and provided two retreat sites and a water dish. Tub lids had holes to permit airflow. Animals were housed for at least five days prior to testing, to allow for acclimation to their surrounds. Water was provided ad libitum, and crickets dusted with calcium and multivitamin powder (Vetafarm Herpevet Multical Dust) were provided every 2–3 days. All animals were fed for the last time two days before the study commenced. Animals were observed daily to ensure health and wellbeing each day during acclimation.

### Formulation of chloramphenicol

Chloramphenicol was formulated in 20%v/v propylene glycol at a dose of 250 μg.mL^− 1^, applied to the ventral pelvis of the toads. The dose and exposure time required were determined by firstly using our previously-described in vitro linear mixed-effect models of absorption in cane toads [35] to predict absorption parameters (flux and permeability coefficient (K_p_)) for chloramphenicol (Table 4). Briefly: the models were developed from in vitro percutaneous absorption data collected for three model chemicals of differing lipophilicity and molecular size, formulated as saturated solutions in ARS. The models require the input of the logP for the chemical to be administered (for chloramphenicol this is 1.14 [56];), and then predict the absorption parameter based on the logP of the chemical to be administered and the site of application (dorsal, ventral thoracic or ventral pelvis). Predictions from these models were made using the predict function in base R [57].

**Table 4:** Predicted flux and K_p_ for chloramphenicol in a solution of ARS through cane toad skin

LogP	Skin Region	Flux (μg/cm^**2**^/h)	K_**p**_ (cm.h^**− 1**^; × 10^**− 3**^)
		In vitro (predicted)	In vitro (predicted)
1.14	Dorsal	23.406	3.675
1.14	Ventral thoracic	20.764	3.254
1.14	Ventral pelvic	26.006	3.956

.

Following prediction of in vitro absorption parameters for chloramphenicol, the predicted flux was adjusted based on our previous reported finding that the in vitro models overestimated in vivo absorption for the model chemicals in frog skin [35]. The extent of difference between these parameters differ based on the model chemical’s logP. As flux is inversely related to logP in ventral pelvic cane toad skin, the likely impact of this difference in chloramphenicol can be estimated (see supplementary materials).

Duration of exposure was calculated based on the time to reach a target serum concentration of 12.5 μg/ml (i.e., the MIC of chloramphenicol for *Batrachochytrium dendrobatidis* [37]), predicted in vivo flux for chloramphenicol, the animal’s estimated blood volume and the surface area of skin exposed to the formulation (for details, see supplementary materials). As reduced exposure time would be of benefit in a clinical situation, the addition of 20%v/v PG as a penetration enhancer was considered to increase absorption rate. Our previous study reported a relationship between logP and the enhancement ratio (ER) for model chemicals when applied to the ventral pelvis in cane toads [36]. Thus, it is expected that the absorption rate from this formulation will increase according to this relationship. Further details of the process undertaken in formulating chloramphenicol as described are available in the supplementary materials.

As the secondary aim of this study was to provide dosing guidelines for use of chloramphenicol in *Batrachochytrium dendrobatidis* infection, the decision was made to continue dosing past the expected time to attain therapeutic levels, to determine if levels would continue to increase. The study therefore bathed the animals for a period of 6 h, hereafter termed the “exposure” period, after which the bath was ceased, animals rinsed to remove residual drug solution, and serum levels taken for a further 18 h to determine drug persistence and/or elimination in these animals (“elimination period”).

### Study design

Prior to study commencement, each animal was rinsed with ARS and individual animal weights were recorded. Following weighing, animals were housed in individual plastic containers for the duration of the trial. These containers restricted movement of the animals, ensuring that drug solution exposure was mainly to the ventral pelvic skin. Animals were randomly allocated to a sampling time using random number generation software.

25 mL of the chloramphenicol solution (containing 6.341 mg chloramphenicol) was transferred to individual plastic zip-lock bags. Each animal was transferred from their individual container into a bag containing chloramphenicol solution, and then rehoused in their individual plastic container within the bag. Each animal received the same dose (~ 250 μg.mL^− 1^), resulting in an approximate dose per animal of 62.6 μg/g.

The study was divided into two phases: (1) exposure phase (t = 0 to t = 6 h), and (2) elimination phase (t = 6 to t = 24 h). During the exposure phase, animals were exposed to the drug solution, with samples being taken at t = 0.25, 0.5, 1, 1.5, 2, 3, 4, 6 h. At t = 6 h, all remaining animals were rinsed and returned to their individual plastic containers for the elimination phase. Samples were then taken at t = 8, 10, 12, 18, and 24 h. Four animals were sacrificed at each sampling time, as per the OECD guidelines for in vivo dermal absorption testing [58]. Three additional animals served as controls and were bathed in 20% v/v PG solution alone following the same two phases. These animals were sacrificed at t = 0, 6 and 24 h. Animals were closely observed throughout the study.

At each sampling time, four animals were removed from the chloramphenicol solution and rinsed in 25 mL of fresh purified water. Rinsing solution and the remaining chloramphenicol solution were retained, total volume determined to ascertain if urine had been produced during the experiment, and a sample analysed for drug content.

Immediately following rinsing, blood samples were taken via cardiac puncture as previously described [35]. Briefly: each animal was anaesthetised by intracoelomic injection of 400 mg/kg MS-222 [55]. A deep plane of anaesthesia was achieved within 2 min of MS-222 administration, following which the thoracic cavity was opened and 1 mL of cardiac blood removed via heparinised capillary tube. Immediately following sample collection, animals were chemically euthanized by prolonged bathing in buffered 0.2%w/v MS-222 solution [54]. Samples were allowed to clot, then centrifuged at 12,000 RCF for 10 min. 0.25 mL aliquots of serum were transferred to clean Eppendorf tubes, and stored at − 80 °C until analysis.

### Sample extraction

The extraction process used was based on the method described by Greiner-Sosanko et al. [59]. Prior to extraction, 40 μL of IS solution was added to each thawed aliquot of serum and vortexed for 30 s. To each serum sample was then added 1.5 mL of 10 mmol sodium hydroxide and 4 mL of ethyl acetate. The resultant mixture was immediately vortexed for 60 s, and centrifuged at 4500 RCF for 5 min at 25 °C. The organic layer was then transferred to a clean glass tube. The extraction process was repeated a second time, as preliminary studies showed only 88% recovery following one extraction cycle. Organic supernatants were combined, and gently dried under nitrogen gas at 40 °C. Samples were then reconstituted with 1 mL of mobile phase, vortexed, and centrifuged for 5 min. The clear supernatant, in a clean sample vial, was transferred immediately to the laboratory for analysis. Preliminary studies indicated that 97% of chloramphenicol was extracted following two extraction cycles.

### Ultra-high-performance liquid chromatography (UHPLC)

Analysis of serum and urine samples (applied without any extraction) was performed on a Shimadzu UHPLC Nexera X2, with an SPD-M30A Diode Array Detector, and post-run analysis was performed using Labsolutions 5.89 (Shimadzu). The HPLC method used has been reported previously [35]. Separation was carried out by gradient elution, using an Applied Biosystems SPHERI-5 5 Micron ODS column (250 × 4.6 mm) at 38 °C. The mobile phase was of 0.2% v/v acetic acid in water: methanol, increasing from 50 to 85% methanol over the first 12 min, and remaining at 85% methanol for a further 3 min, for a total run time of 15 min. The flow rate was 1 mL.min^− 1^, and injection volume was 10 μL. Quantification was at 242 nm, and all samples were analysed in duplicate.

Under the conditions described, the retention times of chloramphenicol and IS were 4.28 and 8.07 min, respectively. A standard curve was prepared by spiking chloramphenicol and IS into mobile phase, as preliminary studies in pooled blank toad serum showed no matrix effects. The method exhibited good linearity over 0.25–175 μg.mL^− 1^, with r^2^ > 0.999 for all runs. The limit of quantification was 0.25 μg.mL^− 1^.

### Data analysis and statistics

Data analysis and statistics were performed in R [57]. Predictions from models were made using the predict function in base R. A naïve pooled approach was used and pharmacokinetic parameters calculated using standard noncompartmental methods in the PKNCA package [60]. T_max_ (time to maximum plasma concentration) and C_max_ (maximum peak plasma concentration) were taken from the observed data. Area under the concentration-time curve up to 24 h (AUC_0–last_) and area under the concentration-time curve with extrapolation to infinity (AUC_0–∞_) were calculated using the linear trapezoidal method for ascending concentrations and the logarithmic trapezoidal method for descending concentrations. The terminal elimination rate constant (k_el_) was determined from the slope of the terminal portion of the elimination curve using at least three sampling points.

## Supplementary Information

**Additional file 1.**

## Data Availability

The datasets supporting the conclusions of this article are available in the Tropical Data Hub repository (10.25903/5d4d00bb0d310; 10.25903/5d4a6ff6bae14; 10.25903/5d4a733601e97). Requests for material should be made to the corresponding author.

## Abbreviations

ARS: Amphibian Ringers Solution
AUC_0–last_: Area under the concentration-time curve up to 24 h
AUC_0–∞_: Area under the concentration-time curve with extrapolation to infinity
C_max_: Maximum observed plasma concentration
ER: Enhancement ratio
FOD: Factor-of-difference
IS: Internal standard
k_el_: Terminal elimination rate constant
K_p_: Permeability coefficient
MIC: Minimum inhibitory concentration
PG: Propylene glycol
SD: Standard deviation
T_max_: Time to maximum plasma concentration
UHPLC: Ultra-high-performance liquid chromatography
