# Correction to: Predicting in vivo absorption of chloramphenicol in frogs using in vitro percutaneous absorption data

**DOI:** 10.1186/s12917-021-02860-7

**Published:** 2021-04-14

**Authors:** Victoria K. Llewelyn, Lee Berger, Beverley D. Glass

**Affiliations:** 1 Pharmacy, College of Medicine and Dentistry, James Cook University, Townsville, Australia; 2 College of Nursing and Health Sciences, Flinders University, Adelaide, Australia; 3 One Health Research Group, Melbourne Veterinary School, University of Melbourne, Werribee, Australia

**Correction to: BMC Vet Res 17, 57 (2021)**

**https://doi.org/10.1186/s12917-021-02765-5**

The original article [1] contained an error mistakenly caused by the production team whereby the footer for Table 4 was omitted from the published article. This has since been re-introduced.
