# Supplementary material for: Predicting in vivo absorption of chloramphenicol in frogs using in vitro percutaneous absorption data
Source: BMC Vet Res. 2021 Jan 28;17:57. doi: 10.1186/s12917-021-02765-5 (PMC7842057; doi:10.1186/s12917-021-02765-5)
Supplement: Supplementary file 1 — Additional file 1. [file 12917_2021_2765_MOESM1_ESM.docx]

**Considerations in determining the appropriate dose and dosing interval for chloramphenicol**

***Adjusting predicted levels (from in vitro-based models) based on in vivo findings for model chemicals***

The in vitro models overestimated the in vivo absorption for the model chemicals in frog skin, with the extent of difference between these parameters differing based on the model chemical’s logP [1]. The relationship between flux and logP is inverse linear [2]; and so we can predict the likely effect of this difference for chloramphenicol. Our previous study [1] reported the factor-of-difference (FOD) between in vitro predicted and in vivo measured absorption parameters was ~4.6-fold (flux) and 4.9-fold (K_p_) for benzoic acid, a chemical with a higher logP than chloramphenicol (logP of 1.87), whereas caffeine (logP –0.07) had a FOD of 12.3 for both parameters [1]. From this relationship, the FOD for chloramphenicol predicted in vitro compared to likely in vivo can be estimated at 7.6-fold for flux, and 7.7-fold for K_p_.

***Estimating dose and exposure time***

An estimate of total drug required and duration of exposure for this study can be made from: the required total serum concentration, predicted in vivo flux for chloramphenicol, the animal’s estimated blood volume and skin exposure area (i.e., ventral pelvic surface area). Standard physiological values for cane toads (Table A) were used for all calculations. For all calculations, animal weight is assumed to be 100 grams.

Table A: Calculation of blood volume, total and pelvic ventral surface area in cane toads

| **Parameter** | **Calculated as** | **Value for 100 g toad** | **Reference** |
| --- | --- | --- | --- |
| Blood volume | 0.0897*weight | 8.97 mL | [3] |
| Pelvic surface area | 1.15*[weight^0.559^] | 15.09 cm^2^ | [4] |
| Total surface area | 9.9*[weight^0.56^] | 130.51 cm^2^ | [5] |

weight is total animal body weight in grams

*Total serum concentration*

The total serum concentration required in vivo for 12.5 µg.mL^-1^ (the MIC for chloramphenicol in *Bd*) to be reached was estimated as the target serum concentration multiplied by the blood volume, i.e., 12.5*8.97 = 112.125 µg.

*Predicted in vivo flux*

As the in vivo flux of chloramphenicol (formulated in ARS) through the ventral pelvis was predicted to be 3.444 µg/cm^2^/h (Table 5), the flux of chloramphenicol in a 100 gram toad through the ventral pelvis would be 15.09 cm^2^*3.444 µg/cm^2^/h = 51.970 µg/cm^2^/h.

*Time to target concentration (i.e., minimum exposure time required)*

It would therefore take 112.125/51.970 = 2.157 hours to reach the serum levels required.

***Adjustments due to inclusion of propylene glycol in the formulation***

To optimise absorption, we formulated chloramphenicol in 20% v/v PG. We previously reported that PG effectively enhanced penetration of moderately- and highly-lipophilic chemicals through cane toad skin, with enhancement ratios (ER) of 3.95 for benzoic acid and 2.28 for ibuprofen [6] when applied to the pelvic ventrum. The relationship between logP and ER for these chemicals through the ventral pelvis, when formulated in 20% v/v PG, can be described by the equation y=–0.587x^2^ + 2.6317x + 1.0844 (r^2^=1). This equation was used to estimate the ER of chloramphenicol in 20% v/v PG through ventral pelvic skin (ER = 3.32).

If ~3.2-fold faster absorption can be expected by formulating chloramphenicol in 20% v/v PG, the required therapeutic blood levels may be achieved within ~40 minutes.

***Determination of dose to be administered***

The provided calculations are based on a saturated drug solution being administered. Such a solution for chloramphenicol in water would contain ~2500 µg.mL^-1^ chloramphenicol. Using such a concentrated solution would be both costly and impracticable. As the blood level required to attain the target concentration is only 12.5 µg.mL^-1^, and dosing solutions are considered adequately “saturated” in percutaneous absorption studies provided there is a 10-fold excess of drug in the dosing solution compared to the maximum concentration expected to be absorbed [7], a decision was made to revise this value down. Based on the target concentration, a 10-fold excess would be a solution containing 125 µg.mL^-1^ of chloramphenicol. However, Holden et al. [8] reported that a 200 µg.mL^-1^ solution of chloramphenicol reduced infection load, but did not clear, *Bd* infection in *R. sphenocephala*, providing motivation for a dose of 250 µg.mL^-1^ in the current study.

NOTE: Some assumptions must be made when using the above data — that is, that the drug is not widely distributed and that there is no protein binding. While the pharmacokinetics of chloramphenicol in toads is not known, its protein binding, solubility and distribution in mammals suggests that neither of these assumptions are likely to be true [9]. However, as this is a preliminary study, some assumptions are necessary.

**References**

1. Llewelyn VK, Berger L, Glass BD: Can models of percutaneous absorption based on in vitro data in frogs predict in vivo absorption? PLoS One 2020;15(7):e0235737.

2. Llewelyn VK, Berger L, Glass BD: Effects of skin region and relative lipophilicity on percutaneous absorption in the toad *Rhinella marina*. Environ Toxicol Chem 2019;38(2):361-7.

3. Baustian M: The contribution of lymphatic pathways during recovery from hemorrhage in the toad *Bufo marinus*. Physiol Zool 1988;61(6):555-63.

4. Tracy CR: A model of the dynamic exchanges of water and energy between a terrestrial amphibian and its environment. Ecol Monogr 1976;46:293–326.

5. McClanahan Jr. L, Baldwin R: Rate of water uptake through the integument of the desert toad, *Bufo punctatus*. Comp Biochem Physiol 1969;28(1):381-9.

6. Llewelyn VK, Berger L, Glass BD: Permeability of frog skin to chemicals: effect of penetration enhancers. Heliyon 2019;5(8):e02127.

7. Organisation for Economic Co-operation and Development. Guidance notes on dermal absorption. 156 ed. Paris: OECD Environment, Health and Safety Publications; 2011.

8. Holden WM, Ebert AR, Canning PF, Rollins-Smith LA: Evaluation of amphotericin b and chloramphenicol as alternative drugs for treatment of chytridiomycosis and their impacts on innate skin defenses. Appl Environ Microbiol 2014;80(13):4034-41.

9. Papich MG. Chloramphenicol and Derivatives, Macrolides, Lincosamides, and Miscellaneous Antimicrobials. In: Riviere JE, Papich MG, editors. Veterinary Pharmacology and Therapeutics. 10 ed. Hoboken, NJ: John Wiley & Sons, Incorporated; 2018. p. 903-52.
